# Early-life thermal stress and mid-growth light intensity as key environmental predictors of lameness in commercial Cobb 500 broilers under tropical closed-house production systems

**DOI:** 10.14202/vetworld.2026.2233-2251

**Published:** 2026-05-29

**Authors:** Parita Noisamran, Wiriya Loongyai, Choawit Rakangthong, Chaiyapoom Bunchasak

**Affiliations:** Department of Animal Science, Faculty of Agriculture, Kasetsart University, Bangkok, Thailand

**Keywords:** ammonia concentration, broiler chickens, environmental management, lameness, light intensity, temperature fluctuation, Thailand, welfare

## Abstract

**Background and Aim:**

Lameness is a major welfare and economic concern in fast-growing broiler chickens, particularly under tropical intensive production systems where environmental stressors are pronounced. Despite its multifactorial etiology, large-scale field evidence quantifying the combined effects of housing microclimate on lameness in Southeast Asian closed-house systems remains limited. This study aimed to identify age-specific environmental predictors of lameness in commercial Cobb 500 broilers raised under tropical closed-house conditions.

**Materials and Methods:**

An observational study was conducted using data from 296 broiler flocks (11.45 million birds) across five commercial farms in Eastern Thailand during 2023. Lameness incidence was derived from culling records. Environmental parameters included temperature, relative humidity, temperature–humidity index (THI), light intensity, and ammonia concentration. Statistical analyses comprised descriptive statistics, General Linear Model, Pearson’s correlation, and multivariable regression, with assessment of multicollinearity.

**Results:**

The median lameness incidence was 1.00% (interquartile range: 0.76–1.38%), with significant variation among farms (p < 0.0001). Early-life thermal conditions were strongly associated with lameness, with day 1 maximum temperature showing a positive correlation (r = 0.54, p < 0.0001). Diurnal temperature variation on day 7 exhibited a negative association (r = −0.36, p < 0.0001), indicating the importance of thermal stability. Light intensity demonstrated the strongest association during mid-growth, particularly on day 14 (r = 0.56, p < 0.0001). Ammonia concentration became a significant factor during late production stages (day 28 onward; r = 0.31–0.41, p < 0.0001). THI values consistently exceeded recommended thresholds, indicating sustained thermal stress. The final multivariable model explained 42.9% of the variation in lameness incidence (adjusted R² = 0.416), identifying day 7 diurnal temperature variation and day 14 light intensity as independent predictors.

**Conclusion:**

Lameness in tropical broiler systems is strongly influenced by age-specific environmental conditions. Early thermal stress and excessive mid-growth light intensity emerged as critical predictors, while late-stage ammonia accumulation further increased risk. These findings highlight the importance of precise microclimate management, particularly during early and mid-growth phases, to improve broiler welfare and reduce economic losses in tropical closed-house production systems.

## INTRODUCTION

In the past five decades, intensive genetic selection has substantially increased broiler growth rates, with modern commercial strains achieving daily weight gains of 65–100 g, more than three times those reported in the 1970s [1, 2]. Cobb 500 broilers typically reach approximately 2 kg of body weight at around 31 days of age, reflecting a high growth rate [3]. While this genetic progress has markedly improved production efficiency, it has concurrently increased susceptibility to metabolic disturbances, musculoskeletal disorders, and compromised bone integrity. Consequently, skeletal and locomotor disorders, particularly lameness, remain among the most critical welfare and economic challenges in the global broiler industry. Recent estimates indicate that leg disorders contribute to 15–30% of production losses due to impaired growth, increased culling, carcass condemnation, and reduced welfare, depending on the production system and genetic line [4, 5]. Birds affected by lameness experience reduced mobility, behavioral restriction, and chronic pain, raising major welfare concerns and negatively influencing consumer perception and compliance with international animal welfare standards [6, 7].

Lameness in broilers is commonly evaluated using gait scoring systems, most notably the Bristol gait scoring system (scores 0–5) [8] and the U.S. gait scoring system (scores 0–2) [9]. In commercial practice, a lameness incidence below 2% is generally considered acceptable, whereas higher values often indicate compromised management, environmental stress, or genetic predisposition [10]. Among the underlying pathological conditions, bacterial chondronecrosis with osteomyelitis (BCO) is one of the most prevalent causes of severe lameness in fast-growing broilers, particularly under high mechanical loads and microbial challenges [11]. Lameness development is multifactorial, involving both intrinsic factors (e.g., genetics and growth rate) and extrinsic factors, among which housing microclimate plays a central role in determining broiler leg health and welfare [5].

Environmental conditions within broiler houses, particularly temperature, light intensity, and air quality, are increasingly recognized as critical extrinsic drivers of locomotor health. Recent studies conducted in tropical and subtropical production systems have demonstrated that microclimate variables, such as ambient temperature, humidity, and thermal indices, strongly influence broiler welfare and performance [12–15]. However, most existing research has evaluated these environmental factors independently, despite their frequent interaction under commercial conditions.

Despite the growing body of evidence on environmental influences on broiler welfare, several critical gaps remain. First, existing studies have largely focused on isolated environmental factors rather than evaluating their combined and interactive effects under commercial production conditions. Second, there is a lack of large-scale, multi-farm field studies conducted under tropical closed-house systems, where environmental stressors are more pronounced and management practices differ from temperate or open-sided systems. Third, limited information is available regarding age-specific susceptibility to environmental stressors, particularly during key developmental phases when skeletal growth and metabolic demands are highest. Furthermore, no published study has simultaneously quantified the integrated effects of temperature patterns, light intensity, and ammonia concentration on lameness incidence in commercial broiler production systems in Southeast Asia. This lack of comprehensive, real-world data limits the ability to develop precise, stage-specific management strategies to mitigate lameness.

Therefore, the aim of this study was to evaluate the combined influence of key environmental variables, including temperature, temperature-related indices, light intensity, and ammonia concentration, on lameness incidence in commercial Cobb 500 broilers raised under tropical closed-house conditions in Eastern Thailand. Specifically, this study sought to identify age-specific environmental predictors and critical risk windows associated with lameness development, using a large-scale, multi-farm dataset under standardized commercial management. The findings are intended to provide practical, evidence-based insights to improve microclimate management, enhance broiler welfare, and reduce economic losses in tropical poultry production systems.

## MATERIALS AND METHODS

### Ethical approval

This study used retrospective and prospective field data collected from commercial broiler farms operating under standard industry management practices. No experimental manipulation, invasive procedures, or handling of live animals was performed specifically for research purposes. Therefore, formal approval from an Institutional Animal Care and Use Committee (IACUC) or equivalent ethical review board was not required.

All procedures complied with the national livestock and animal welfare regulations of Thailand and adhered to internationally recognized animal welfare standards, including the principles of the Five Freedoms. The participating farms were certified under the LRQA Farm First assurance scheme, which ensures compliance with global animal welfare, biosecurity, and management guidelines.

Routine flock management, including health monitoring, culling decisions, and environmental control, was performed by trained farm personnel under the supervision of licensed veterinarians employed by the integrator company. Birds identified with moderate to severe lameness were humanely removed from the flock in accordance with established welfare protocols to prevent unnecessary suffering.

Data used in this study were obtained exclusively from routine farm records and automated environmental monitoring systems. No additional interventions were introduced for research purposes. Farm identities were anonymized (Farms 1–5) to ensure confidentiality and to comply with data protection policies agreed upon with the participating commercial entities.

The study design and data usage were reviewed and approved internally by the Department of Animal Science, Faculty of Agriculture, Kasetsart University, Bangkok, Thailand, to ensure compliance with ethical standards for the use of production data in research. The study adhered to accepted ethical principles for observational research involving animals and did not compromise animal welfare at any stage.

### Study period and location

This observational study was conducted between January and December 2023 in the eastern region of Thailand.

### Study design

Data on environmental conditions and poultry performance were gathered using Aviapp® (Huvepharma, Bangkok, Thailand), a cloud-based digital tool designed for monitoring poultry production. While Aviapp® can also track flock health indicators, this study focused solely on analyzing environmental factors (such as temperature, light intensity, and ammonia levels) and performance-related data. Although Aviapp allows direct mobile data entry, this study used a structured protocol. Farm workers recorded information manually, which house supervisors then entered into Aviapp®. The farm veterinarian and manager reviewed the data before final submission to the cloud system, ensuring multi-level quality control and minimizing errors.

At first, 296 commercial broiler flocks were evaluated. To maintain consistent data for statistical analysis, any flock with missing lameness records or essential environmental details was removed from the study. However, no flocks were excluded due to performance issues, disease status, or environmental conditions, which helped reduce selection bias.

The investigation covered five commercial broiler farms operating under standardized intensive production systems. All farms were managed by the same integrator and followed uniform feeding, health, and biosecurity protocols to ensure consistency in management. Each farm utilized closed-house systems equipped with double flooring and evaporative cooling mechanisms to maintain optimal thermal comfort. Houses were tunnel-ventilated under negative pressure, with wet-pad panels automatically controlled for temperature and humidity. House dimensions were 120 × 22.5 m for Farm 1, 125 × 36 m for Farm 2, 125 × 22.5 m for Farm 3, and 125 × 45 m for Farms 4 and 5. Final stocking density at slaughter was similar across farms, ranging from 25.5 to 26.54 kg/m², minimizing potential confounding effects on lameness outcomes. All houses used fresh rice hulls as litter, fully replaced each cycle, with mean litter allocation ranging from 5.17 to 5.98 kg/bird across farms.

A total of 296 flocks (representing 11,449,800 Cobb 500 broiler chickens) were included. The flock distribution by farm was as follows: Farm 1: 1,662,400 broilers (60 flocks); Farm 2: 2,772,500 broilers (60 flocks); Farm 3: 2,552,600 broilers (92 flocks); Farm 4: 1,665,500 broilers (36 flocks); Farm 5: 2,796,800 broilers (48 flocks). Broiler flocks on five farms included male, female, and mixed-sex groups. Males had higher lameness rates than females (1.18% vs. 0.96%, p = 0.0002). Because sex was closely linked to growth rate and body weight and was unevenly distributed, it was excluded from the final multivariable regression to avoid multicollinearity and was kept only for descriptive analysis.

All broiler chicks were sourced from multiple commercial hatcheries within the same integrator system using parent stock from its breeder farms. Mean breeder age was similar across farms (approximately 39.22–41.16 weeks). Because hatchery sources and parent stock origins overlapped, hatchery-specific analysis was not feasible in this study.

All diets were centrally manufactured at the same commercial feed mill and formulated (corn–soybean meal-based) to meet or exceed Cobb 500 nutritional recommendations. A four-phase feeding program was applied uniformly across farms: Starter 1 (crumble) from 1 to 15 days of age, Starter 2 (crumble) from 14 to 22 days, Grower (pellet) from 23 days to 7 days before slaughter, and Finisher (pellet) during the last 7 days before slaughter. Standardized levels of calcium, phosphorus, and vitamin D were applied across all farms, and no additional mineral or vitamin supplementation was used at the farm level. This centralized feed production ensured nutritional uniformity among flocks. Vaccination schedules and medication protocols were standardized across farms in accordance with the integrator’s veterinary guidelines.

Lighting programs were standardized across farms but implemented with age-dependent adjustments. All farms provided near-continuous light (23–24 h/day) during the first week after placement, followed by a gradual reduction in photoperiod during the growing period. From approximately 2 to 3 weeks of age, the daily light duration was reduced to 18–20 h with a consolidated dark period to promote rest. Thereafter, birds were maintained under 16–18 h of light per day until slaughter. Although the overall structure of the lighting program was uniform, the exact timing of light-on and light-off cycles varied slightly among farms (e.g., lights-on at 21:00–22:00 and dark periods ranging from 2 to 7 h/day depending on farm and age).

Ventilation and temperature management were controlled automatically in all houses using a Temp 1200 Plus controller. Target temperatures were set at 32–33°C at placement and gradually decreased with age to 26–27°C by 3–4 weeks and to 21–22°C by slaughter age. Evaporative cooling pads were activated when the house temperature exceeded the target by approximately 0.3–1.0°C. Minimum and maximum ventilation were achieved through staged operation of tunnel fans, ranging from 1–3 fans at minimum to 17–35 fans at maximum, depending on farm and house size. Heater set points were typically maintained 0.1–0.5°C below the target temperature during brooding.

While all farms adhered to the same integrator guidelines and used the same controller model, specific environmental settings varied slightly among farms. Differences were observed in cooling-pad differential settings, heater offsets, and fan staging patterns, primarily due to variations in house dimensions. Larger houses (Farms 4 and 5) operated a greater number of tunnel fans compared with smaller houses (Farms 1–3), resulting in farm-specific ventilation capacity despite a standardized overall microclimate management strategy.

### Assessment of lameness

Daily flock inspections were conducted by trained farm personnel as part of standard management routines. Birds exhibiting visible lameness, such as limping, reluctance to move, or an abnormal gait, were manually captured and removed from the flock. The number of lame birds culled per day was recorded at the flock level and used to calculate the percentage of lameness using the following equation:

Lameness (%) = (Number of lame birds culled / Total birds in flock) × 100

Lameness was assessed using a standardized commercial visual scoring system adapted from established gait scoring methods. Birds showing moderate to severe gait impairment (approximately Bristol gait score ≥3) were culled for welfare reasons. Farm personnel received annual training from company veterinarians using reference images and videos, with periodic supervisory checks to reduce inter-observer variability.

Only birds with clear locomotor impairments leading to culling were classified as lame to ensure accuracy and reduce observer bias. Subclinical or borderline cases were excluded unless removal was required for welfare reasons.

### Environmental temperature measurement

Ambient temperature and relative humidity (RH) were recorded using integrated digital sensors of the Temp 1200 Plus controller system installed at the central zone of each house at bird level. One sensor per house was used as part of the automated climate control system and was routinely calibrated by technical staff and positioned at bird level (approximately 30 cm above the floor). Although multi-point monitoring was not available, the central location was considered representative of average flock exposure under uniform tunnel-ventilated conditions.

Temperature was recorded in °C on days 1, 7, 14, 21, 28, 35, and the day before slaughter. For each sampling day, the daily maximum (Tmax) and daily minimum (Tmin) temperatures were recorded, and the diurnal temperature variation (ΔT) was calculated as:

ΔT = Tmax − Tmin

Relative humidity (RH, %) was recorded from the same house controller sensors at the corresponding sampling days. Descriptive statistics of RH at the time of the highest and lowest daily temperatures are provided in Supplementary Table 2. These temperature–humidity data were used to compute the temperature–humidity index (THI).

THI was calculated according to the following equation:

THI = (0.8 × T) + [RH/100 × (T − 14.4)] + 46.4

where T represents ambient temperature (°C) and RH represents relative humidity (%). For THI at high temperature, T = Tmax; for THI at low temperature, T = Tmin, using the corresponding RH recorded at the same time point.

### Light intensity measurement

Light intensity (lux) was measured with a calibrated handheld digital lux meter between 09:00 and 10:00 h during the active photoperiod, when the artificial lighting systems were fully operational. Measurements were performed at bird level (approximately 30 cm above the floor) at the central location of the house to avoid edge or reflection bias.

Readings were obtained on days 1, 7, 14, 21, 28, 35, and the day before slaughter to represent changes in illumination throughout the production cycle. Light intensity was measured at two standardized locations in each house (central and rear zones). Because rear measurements were influenced by external light entering through ventilation openings and fan inlets, only central-zone readings were used for statistical analysis to minimize measurement bias.

### Ammonia concentration measurement

Ammonia concentrations (NH_3_, ppm) were determined with a portable gas detector (ToxiRAE Pro, RAE Systems, Honeywell, San Jose, USA). The device was calibrated at the factory and then recalibrated annually by qualified technicians. Per the manufacturer’s guidelines, the instrument’s accuracy is within approximately ±2 ppm or ±10% of the reading, whichever value is higher.

Measurements were taken once per day at a central bird-level location (≈30 cm above the floor) between 09:00 and 10:00 h, reflecting typical exposure under tunnel ventilation. These values were used as the daily flock-level ammonia concentration. Instruments were calibrated before use. Multi-point sampling was not routinely conducted, which is a limitation. Ammonia levels above 10–15 ppm exceeded recommended welfare thresholds for broilers [3].

### Statistical analysis

All statistical analyses were conducted using raw lameness percentages (0–100%) and performed using SAS University Edition (SAS Institute Inc., Cary, NC, USA) following the SAS/STAT® guidelines [16]. Prior to analysis of variance (ANOVA), lameness percentage was tested for normality using the Shapiro–Wilk test and for homogeneity of variance using Levene’s test. Analyses were performed using raw percentages (0–100%), and raw means are reported for interpretation.

A General Linear Model (GLM) was used to evaluate differences in lameness incidence among farms. In this model, the dependent variable was lameness percentage, and the fixed independent factor was Farm (five levels). The GLM model was expressed as:

*Y_ij_* = *μ* + *τ_i_* + *ε_ij_*

where *Y_ij_* is the observation for treatment *i*, *μ* is the overall mean, *τ_i_* is the treatment effect (farm), and *ε_ij_* is the random error term.

When significant effects were detected (p < 0.05), mean comparisons were performed using Duncan’s New Multiple Range Test (DMRT), applied as an exploratory post hoc comparison for descriptive purposes in a commercial field dataset.

Pearson’s correlation analysis was applied to assess the relationships between lameness incidence (dependent variable) and environmental parameters (independent variables: maximum temperature, minimum temperature, diurnal temperature variation, light intensity, and ammonia concentration). The Pearson correlation coefficient (r) was calculated as:


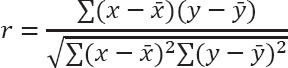


The significance of each correlation coefficient was tested using a *t*-test:


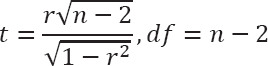


A p < 0.05 was considered statistically significant, and < 0.01 was considered highly significant. The interpretation of correlation strength followed the criteria of Best method [17] as shown in Table 1.

**Table 1:** Rule of thumb for interpreting the size of a Pearson’s correlation coefficient [17].

Size of correlation	Interpretation
0.81 to 1.00 (−0.81 to −1.00)	Very high positive (negative) correlation
0.51 to 0.80 (−0.51 to −0.80)	Moderate positive (negative) correlation
0.21 to 0.50 (−0.21 to −0.50)	Low positive (negative) correlation
0.01 to 0.20 (−0.01 to −0.20)	Very low positive (negative) correlation
0.00	Negligible correlation

To explore potential environmental predictors of lameness, a stepwise multiple linear regression was first performed using PROC REG in SAS. The dependent variable was flock-level lameness percentage (0–100%). Normality and homogeneity assumptions were evaluated prior to analysis, and the candidate independent variables included the highest and lowest temperatures, diurnal temperature difference, light intensity, and ammonia concentration on each measurement day. Variables were entered into and removed from the model based on statistical criteria (entry and stay p-value < 0.15), partial R², Mallows’ C(p), and biological plausibility.

A stepwise regression procedure was used to screen variables for candidate predictors. Due to multicollinearity and biological considerations, the stepwise output was not used as the final model but served only for variable preselection (Supplementary Table 3).

Based on a priori biological relevance and the correlation results, a final multivariable regression model was then fitted with five predictors: the highest temperature on day 1, the diurnal temperature difference on day 7, the light intensity on day 14, and the ammonia concentrations on days 28 and 35. Model fit was assessed using the coefficient of determination (R² and adjusted R²), F-test for overall significance, root mean square error (RMSE), and examination of residual plots to verify linear model assumptions.

For the multiple regression model, multicollinearity was assessed using variance inflation factors (VIF), with values <5 indicating acceptable levels. Residual independence was evaluated using the Durbin–Watson statistic (0.98), suggesting mild positive autocorrelation. Residual normality was examined using Q–Q plots, which largely followed the theoretical normal distribution with minor upper-tail deviations that were not considered to meaningfully affect model validity.

### Power of analysis

A post hoc power analysis was conducted for the primary outcome (% lameness) comparing farms. Because farm differences were analyzed using a General Linear Model (GLM) within a one-way ANOVA framework, statistical power was calculated using G*Power 3.1 (F tests, fixed effects, omnibus, one-way ANOVA). Based on five groups (farms), a total sample size of 296 flocks, and α = 0.05, the effect size (Cohen’s f = 0.49) indicated a large effect. The achieved statistical power was >0.99, demonstrating that the study had sufficient power to detect differences in % lameness among farms.

Additionally, a retrospective power analysis for correlation analysis was performed using G*Power (two-tailed, α = 0.05). With a total sample size of 296 flocks, the study had >0.99 power to detect a moderate correlation (r = 0.30), indicating sufficient sensitivity to identify meaningful associations between environmental factors and % lameness.

## RESULTS AND DISCUSSION

### Lameness distribution and farm-level variation

**Figure 1:**
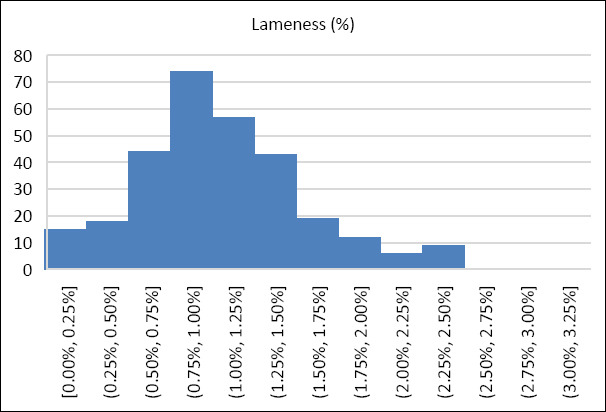
Distribution of lameness incidence (%) across 296 broiler flocks. Figure 1 illustrates the frequency distribution of flock-level lameness incidence across 296 commercial broiler flocks. The histogram shows that most flocks had lameness levels between 0.75% and 1.50%, with the highest frequency around 1.00%. A small proportion of flocks exceeded 2.00%, indicating outliers with elevated lameness incidence. Overall, the distribution shows a slight right skew, suggesting that while most flocks maintained relatively low lameness levels, a subset experienced substantially higher lameness prevalence. This distribution supports the use of parametric statistical analyses after confirmation of approximate normality.

Lameness information from five farms, including N, mean, SD, SEM, min–max, and 95% CI, is presented in Table 2, while the distribution across 296 flocks is illustrated in Figure 1. The median lameness incidence was 1.00%, with the 25th and 75th percentiles at 0.76% and 1.38%, respectively (Supplementary Table 1 and Figure 1). Most flocks exhibited lameness levels below 1.5%, although a small proportion exceeded 2.0%.

**Table 2:** Descriptive statistics of lameness incidence in broiler farms.

Farm	n	Mean (%)	SD	SEM	Min–max	95% CI
1	60	0.68	0.41	0.05	0.14–1.50	0.58–0.79
2	60	1.48	0.47	0.06	0.68–2.51	1.36–1.60
3	92	1.02	0.98	0.04	0.37–2.52	0.94–1.09
4	36	1.56	0.53	0.09	0.57–3.16	1.38–1.74
5	48	0.86	0.20	0.03	0.42–1.25	0.80–0.92

CI = Confidence interval, n = Number of flocks, SD = Standard deviation, SEM = Standard error of the mean.

Compared with previous commercial broiler studies, in which gait abnormalities vary widely due to genetics, management, and environment, this study found a relatively low incidence. While intensively reared flocks often show clinically detectable lameness ranging from a few percent up to over 20%, the levels observed here are low for commercial settings [18].

At the farm level (Table 3), Farm 4 and Farm 2 showed the highest mean lameness rates (1.56 ± 0.09% and 1.48 ± 0.06%, respectively) and did not differ significantly from each other (p > 0.05), but were significantly higher than all other farms (p < 0.05). Farm 3 (1.02 ± 0.04%), Farm 5 (0.86 ± 0.03%), and Farm 1 (0.68 ± 0.05%) had lower mean values, each significantly different per Duncan’s test. Environmental or management factors at Farms 4 and 2 may increase locomotor disorder risk compared to the other farms

**Table 3:** Analysis of variance and Duncan’s multiple range test for lameness percentage by farm.

Farm	n	Mean lameness (%) ± SEM	95% CI
1	60	0.68 ± 0.05ᵃ	0.58–0.79
2	60	1.48 ± 0.06ᵈ	1.36–1.60
3	92	1.02 ± 0.04ᶜ	0.94–1.09
4	36	1.56 ± 0.09ᵈ	1.38–1.74
5	48	0.86 ± 0.03ᵇ	0.80–0.92

ANOVA: F(4, 291) = 45.88, p < 0.0001. Values with different superscript letters within the same column differ significantly (p < 0.05) according to Duncan’s multiple range test. ANOVA = Analysis of variance, CI = Confidence interval, n = Number of flocks, SEM = Standard error of the mean.

Even slight increases in lameness can lower profits by reducing growth, flock uniformity, and increasing mortality, culling, and carcass condemnation. Broiler welfare studies link leg weakness to economic loss and poorer welfare outcomes [18].

Furthermore, the use of a single centralized feed mill with standardized diet formulation minimized nutritional variability among farms, making dietary factors unlikely to confound the associations between environmental conditions and lameness.

### Environmental temperature and lameness relationship

Environmental temperature greatly affects broiler performance and welfare, especially in tropical regions where heat often exceeds comfortable levels. This study assessed daily high, low, and ΔT (Table 4) to quantify thermal load in commercial settings, and correlation analysis revealed significant age-dependent links between temperature and lameness (Table 5). RH, which impacts heat load, was summarized (Supplementary Table 2) and factored into THI calculations (Table 6) to reflect combined temperature and humidity exposure.

**Table 4:** Descriptive statistics of environmental temperature by farm, including the highest temperature, the lowest temperature, and the diurnal temperature.

Day	Farm	n	Mean ± SEM (°C)	SD	Min–max (°C)	95% CI (°C)
**Highest temperature**						
1	1	57	33.52 ± 0.07	0.52	32.5–35.1	33.38–33.66
	2	52	35.47 ± 0.03	0.24	34.5–35.9	35.41–35.54
	3	88	33.98 ± 0.02	0.21	33.6–34.9	33.93–34.02
	4	35	34.83 ± 0.13	0.80	32.9–36.7	34.56–35.10
	5	48	33.97 ± 0.06	0.39	33.0–34.5	33.86–34.08
7	1	58	32.37 ± 0.14	1.03	30.9–36.2	32.10–32.65
	2	60	33.11 ± 0.04	0.30	31.1–33.5	33.04–33.19
	3	92	33.23 ± 0.05	0.44	32.0–33.8	33.14–33.32
	4	36	33.38 ± 0.27	1.59	30.4–39.2	32.84–33.92
	5	48	32.86 ± 0.10	0.66	30.6–33.5	32.67–33.06
14	1	60	31.45 ± 0.10	0.74	29.7–33.2	31.26–31.64
	2	60	32.69 ± 0.13	1.01	30.6–34.2	32.43–32.95
	3	92	31.93 ± 0.09	0.85	29.7–33.6	31.76–32.10
	4	36	31.95 ± 0.16	0.95	30.2–34.4	31.63–32.28
	5	48	31.96 ± 0.08	0.53	30.8–32.8	31.81–32.12
21	1	60	30.79 ± 0.12	0.92	28.8–32.8	30.55–31.03
	2	60	31.74 ± 0.09	0.73	29.4–33.1	31.55–31.93
	3	92	30.56 ± 0.15	1.44	26.0–33.3	30.26–30.86
	4	36	30.66 ± 0.24	1.43	28.5–33.8	30.18–31.15
	5	48	31.11 ± 0.07	0.50	29.9–31.7	30.96–31.25
28	1	60	30.23 ± 0.18	1.40	24.2–32.8	29.87–30.59
	2	60	30.57 ± 0.07	0.58	29.7–33.2	30.42–30.72
	3	92	29.62 ± 0.15	1.41	25.0–33.4	29.33–29.91
	4	36	29.69 ± 0.18	1.10	27.7–32.8	29.32–30.06
	5	48	30.32 ± 0.07	0.48	29.1–31.8	30.18–30.46
35	1	60	29.95 ± 0.08	0.63	28.6–31.2	29.79–30.11
	2	60	29.75 ± 0.14	1.05	26.1–32.1	29.47–30.02
	3	92	28.79 ± 0.14	1.33	25.1–31.2	28.52–29.07
	4	36	28.88 ± 0.15	0.89	27.0–30.9	28.57–29.18
	5	48	29.01 ± 0.09	0.64	27.1–30.8	28.82–29.19
Slaughter	1	60	28.72 ± 0.05	0.42	28.1–30.1	28.61–28.83
	2	60	29.16 ± 0.14	1.06	26.5–30.8	28.88–29.43
	3	92	27.93 ± 0.08	0.76	26.8–30.3	27.78–28.09
	4	35	28.49 ± 0.20	1.18	26.2–31.7	28.09–28.90
	5	48	28.40 ± 0.11	0.78	26.0–30.6	28.18–28.63
**Lowest temperature**						
1	1	58	31.67 ± 0.09	0.69	29.9–33.2	31.49–31.85
	2	52	33.49 ± 0.13	0.18	33.0–33.9	33.44–33.54
	3	87	33.43 ± 0.04	0.39	31.7–33.8	33.34–33.51
	4	35	33.63 ± 0.16	0.95	31.2–35.4	33.31–33.96
	5	48	32.92 ± 0.08	0.54	31.0–33.5	32.76–33.07
7	1	58	29.58 ± 0.17	1.30	26.5–32.5	29.24–29.92
	2	59	32.23 ± 0.02	0.19	32.0–32.7	32.18–32.28
	3	92	32.28 ± 0.09	0.85	30.2–33.3	32.10–32.46
	4	36	31.74 ± 0.21	1.24	29.2–34.5	31.31–32.17
	5	48	31.56 ± 0.15	1.04	29.0–32.6	31.25–31.86
14	1	58	28.80 ± 0.12	0.89	27.2–31.1	28.57–29.03
	2	60	28.32 ± 0.11	0.89	26.2–29.8	28.09–28.55
	3	92	28.97 ± 0.12	1.13	26.0–31.2	28.73–29.21
	4	33	30.44 ± 0.19	1.08	28.0–32.5	30.06–30.83
	5	48	29.84 ± 0.08	0.53	28.7–30.7	29.68–29.99
21	1	57	28.11 ± 0.15	1.12	25.1–30.5	27.82–28.41
	2	60	26.76 ± 0.12	0.95	23.4–28.0	26.51–27.00
	3	92	27.88 ± 0.13	1.28	22.5–29.6	27.61–28.14
	4	36	28.62 ± 0.14	0.86	26.9–30.8	28.33–28.91
	5	48	29.02 ± 0.06	0.41	27.8–30.0	28.90–29.14
28	1	57	27.30 ± 0.19	1.42	23.3–32.5	26.92–27.67
	2	60	26.27 ± 0.08	0.58	25.1–27.9	26.12–26.42
	3	92	26.82 ± 0.15	1.46	23.0–28.7	26.52–27.12
	4	36	27.97 ± 0.23	1.38	26.0–32.1	27.51–28.43
	5	48	28.07 ± 0.12	0.82	25.0–29.7	27.83–28.31
35	1	58	26.41 ± 0.17	1.31	24.3–30.5	26.06–26.75
	2	60	25.47 ± 0.29	2.25	20.7–28.3	24.88–26.06
	3	92	25.88 ± 0.15	1.42	21.5–27.5	25.58–26.17
	4	36	27.38 ± 0.19	1.14	25.2–30.1	26.99–27.76
	5	48	27.31 ± 0.19	1.32	23.9–28.7	26.93–27.69
Slaughter	1	58	25.76 ± 0.14	1.10	24.2–29.0	25.48–26.05
	2	60	24.99 ± 0.21	1.62	21.8–28.1	24.57–25.41
	3	92	25.96 ± 0.11	1.10	21.1–28.4	25.73–26.18
	4	36	25.81 ± 0.21	1.23	20.3–27.5	25.39–26.23
	5	48	27.10 ± 0.12	0.85	24.2–28.6	26.85–27.34
**Diurnal temperature**						
1	1	57	1.85 ± 0.11	0.86	0.0–4.2	1.62–2.08
	2	52	1.98 ± 0.04	0.31	0.7–2.6	1.90–2.07
	3	87	0.51 ± 0.05	0.48	0.0–2.7	0.41–0.61
	4	34	1.24 ± 0.12	0.72	0.2–3.1	0.99–1.48
	5	48	1.05 ± 0.05	0.35	0.4–2.2	0.95–1.16
7	1	58	2.73 ± 0.14	1.07	0.9–5.5	2.51–3.07
	2	59	0.92 ± 0.03	0.20	0.3–1.3	0.87–0.97
	3	92	0.95 ± 0.07	0.64	0.0–2.5	0.82–1.09
	4	35	1.72 ± 0.22	1.27	0.5–7.6	1.28–2.15
	5	48	1.31 ± 0.09	0.65	0.5–3.4	1.12–1.50
14	1	58	2.68 ± 0.13	0.95	0.4–4.8	2.41–2.96
	2	60	4.38 ± 0.17	1.28	2.0–7.4	4.04–4.71
	3	92	2.98 ± 0.14	1.38	0.3–6.6	2.67–3.25
	4	34	1.49 ± 0.24	1.38	0.4–6.4	0.99–1.98
	5	48	2.13 ± 0.06	0.41	1.0–3.0	2.01–2.25
21	1	57	2.69 ± 0.20	1.50	0.0–7.2	2.27–3.07
	2	60	4.99 ± 0.11	0.88	3.1–7.4	4.76–5.21
	3	92	2.68 ± 0.14	1.39	0.5–5.4	2.40–2.97
	4	36	2.04 ± 0.21	1.25	0.8–5.0	1.62–2.47
	5	48	2.09 ± 0.05	0.37	1.1–3.1	1.98–2.20
28	1	57	2.83 ± 0.16	1.22	0.0–5.5	2.51–3.15
	2	60	4.29 ± 0.11	0.86	2.0–6.7	4.07–4.51
	3	92	2.80 ± 0.14	1.31	0.7–6.3	2.53–3.07
	4	36	1.72 ± 0.18	1.06	0.5–4.5	1.36–2.08
	5	48	2.25 ± 0.10	0.67	1.2–4.4	2.05–2.44
35	1	58	3.53 ± 0.18	1.36	0.0–6.1	3.17–3.89
	2	60	4.28 ± 0.22	1.73	2.0–9.2	3.83–4.73
	3	92	2.92 ± 0.13	1.26	0.3–6.1	2.65–3.18
	4	36	1.50 ± 0.13	0.77	0.4–4.2	1.24–1.76
	5	48	1.70 ± 0.15	1.06	0.5–5.0	1.39–2.01
Slaughter	1	58	2.97 ± 0.13	1.01	0.0–4.9	2.70–3.23
	2	60	4.16 ± 0.15	1.14	2.2–8.6	3.87–4.46
	3	92	1.98 ± 0.11	1.01	0.3–6.3	1.77–2.19
	4	35	2.70 ± 0.29	1.73	0.8–7.8	2.10–3.29
	5	48	1.31 ± 0.09	0.62	0.4–4.1	1.13–1.49

CI = Confidence interval, n = Number of flocks, SD = Standard deviation, SEM = Standard error of the mean.

**Table 5:** Pearson’s correlation coefficients between the highest temperature, the lowest temperature, the diurnal temperature, and the total lameness incidence across all farms and rearing days.

Variable	Day	n	r	95% CI	p-value
Highest temperature	1	280	0.54	0.46 to 0.62	< 0.0001
	7	292	0.13	0.01 to 0.24	0.0271
	14	293	0.38	0.28 to 0.47	< 0.0001
	21	294	0.22	0.10 to 0.32	0.0002
	28	294	−0.03	−0.14 to 0.09	0.6251
	35	294	0.06	−0.05 to 0.17	0.3405
	Slaughter	293	0.09	−0.02 to 0.21	0.1062
Lowest temperature	1	280	0.50	0.41 to 0.59	< 0.0001
	7	290	0.38	0.28 to 0.48	< 0.0001
	14	289	−0.08	−0.18 to 0.04	0.1723
	21	291	−0.12	−0.24 to −0.01	0.0347
	28	291	−0.16	−0.27 to −0.04	0.0085
	35	292	−0.05	−0.16 to 0.06	0.3883
	Slaughter	292	−0.10	−0.21 to 0.02	0.1022
Diurnal temperature	1	278	−0.01	−0.12 to 0.11	0.9155
	7	292	−0.36	−0.46 to −0.26	< 0.0001
	14	290	0.32	0.21 to 0.41	< 0.0001
	21	293	0.26	0.15 to 0.36	< 0.0001
	28	293	0.17	0.05 to 0.28	0.0042
	35	294	0.10	−0.01 to 0.21	0.0807
	Slaughter	293	0.33	0.22 to 0.42	< 0.0001

CI = Confidence interval, n = Number of flocks, r = Pearson’s correlation coefficient.

**Table 6:** Descriptive statistics of temperature–humidity index at the highest and lowest temperatures by farm.

Day	Farm	n	Mean ± SEM (%)	SD	Min–max (%)	95% CI (%)
**Temperature–humidity index at highest temperature**						
1	1	57	85.28 ± 0.27	2.06	79.24–89.73	84.73–85.83
	2	52	85.33 ± 0.05	0.32	84.09–85.98	85.24–85.42
	3	93	83.82 ± 0.41	3.98	73.52–91.14	83.00–84.64
	4	35	84.03 ± 0.25	1.46	80.85–86.82	83.52–84.53
	5	48	83.43 ± 0.23	1.59	81.00–86.88	82.97–83.89
7	1	58	84.64 ± 0.31	2.38	79.45–93.48	84.02–85.27
	2	60	82.55 ± 0.05	0.42	79.86–83.32	82.44–82.65
	3	96	84.76 ± 0.30	2.89	78.37–89.52	84.17–85.35
	4	35	83.22 ± 0.42	2.50	78.03–89.32	82.36–84.08
	5	48	82.85 ± 0.35	2.42	77.52–86.92	82.15–83.55
14	1	60	83.70 ± 0.27	2.11	79.89–88.82	83.16–84.25
	2	60	82.43 ± 0.18	1.37	79.56–84.81	82.07–82.78
	3	96	83.59 ± 0.31	3.02	79.32–90.56	82.98–84.21
	4	35	81.70 ± 0.37	2.20	76.60–85.93	80.94–82.45
	5	48	82.37 ± 0.35	2.45	77.40–86.55	81.66–83.08
21	1	60	83.47 ± 0.24	1.83	80.38–87.22	83.00–83.94
	2	60	81.81 ± 0.18	1.43	77.62–85.50	81.44–82.17
	3	96	81.66 ± 0.32	3.12	75.55–89.04	81.03–82.29
	4	36	81.45 ± 0.53	3.16	77.31–90.43	80.38–82.51
	5	48	81.29 ± 0.25	1.76	76.98–84.68	80.78–81.80
28	1	60	82.15 ± 0.33	2.53	72.41–87.26	81.49–82.80
	2	60	81.09 ± 0.14	1.06	79.02–85.26	80.81–81.36
	3	96	80.94 ± 0.31	3.08	72.62–89.56	80.32–81.57
	4	36	80.11 ± 0.35	2.09	74.58–86.59	79.41–80.82
	5	48	80.88 ± 0.36	2.50	76.01–84.77	80.15–81.61
35	1	60	81.88 ± 0.22	1.72	74.91–84.27	81.43–82.32
	2	60	80.61 ± 0.32	2.50	72.99–85.32	79.96–81.25
	3	96	79.72 ± 0.32	3.18	74.06–86.65	79.07–80.36
	4	36	79.87 ± 0.30	1.80	76.51–83.00	79.26–80.48
	5	48	80.13 ± 0.33	2.30	74.91–83.55	79.47–80.80
Slaughter	1	60	80.45 ± 0.15	1.16	78.18–82.85	80.16–80.75
	2	60	81.19 ± 0.26	1.99	72.85–84.08	80.68–81.71
	3	96	78.86 ± 0.20	1.97	74.84–85.05	78.46–79.26
	4	36	78.86 ± 0.56	3.35	70.23–85.79	77.73–79.99
	5	48	79.80 ± 0.32	2.20	74.04–82.37	79.16–80.43
**Temperature–humidity index at lowest temperature**						
1	1	58	84.25 ± 0.22	1.67	80.15–87.85	83.81–84.69
	2	52	82.84 ± 0.03	0.25	82.30–83.40	82.77–82.91
	3	86	84.52 ± 0.31	2.92	79.42–93.08	83.90–85.15
	4	35	82.39 ± 0.21	1.26	80.22–85.44	81.95–82.82
	5	48	82.22 ± 0.21	1.44	79.89–84.83	81.80–82.63
7	1	58	81.48 ± 0.33	2.54	77.41–88.20	80.81–82.15
	2	60	81.58 ± 0.04	0.34	81.08–82.68	81.49–81.66
	3	96	84.02 ± 0.31	2.99	77.86–88.67	83.41–84.63
	4	36	80.75 ± 0.33	1.97	77.07–87.61	80.08–81.41
	5	48	81.31 ± 0.36	2.46	73.14–85.30	80.59–82.02
14	1	58	80.49 ± 0.32	2.42	74.78–84.36	79.85–81.13
	2	60	77.03 ± 0.18	1.39	73.82–79.51	76.67–77.38
	3	96	79.69 ± 0.21	2.07	74.56–83.04	79.27–80.11
	4	34	79.69 ± 0.47	2.72	69.42–83.52	78.74–80.64
	5	48	79.67 ± 0.30	2.10	75.00–82.64	79.07–80.28
21	1	58	80.07 ± 0.32	2.43	74.48–85.28	79.43–80.71
	2	60	75.59 ± 0.24	1.82	69.70–78.59	75.12–76.06
	3	96	78.43 ± 0.22	2.11	69.61–82.05	78.01–78.86
	4	36	78.47 ± 0.33	1.95	75.16–83.86	77.81–79.13
	5	48	78.98 ± 0.23	1.56	75.05–81.61	78.52–79.43
28	1	58	78.55 ± 0.43	3.24	71.69–88.29	77.70–79.40
	2	60	75.62 ± 0.15	1.20	72.80–78.42	75.31–75.93
	3	94	76.99 ± 0.27	2.62	70.88–81.93	76.46–77.53
	4	36	77.91 ± 0.39	2.31	74.17–85.37	77.13–78.69
	5	48	78.15 ± 0.36	2.46	70.82–81.66	77.44–78.87
35	1	58	77.00 ± 0.35	2.67	71.99–81.62	76.30–77.70
	2	60	74.97 ± 0.51	3.96	66.31–79.47	73.95–75.99
	3	96	75.68 ± 0.25	2.49	68.24–79.88	75.18–76.19
	4	36	77.60 ± 0.27	1.62	75.50–81.34	77.05–78.14
	5	48	77.94 ± 0.40	2.75	71.51–81.99	77.14–78.74
Slaughter	1	58	76.13 ± 0.40	3.05	68.63–82.54	75.32–76.93
	2	60	75.00 ± 0.34	2.65	69.80–80.00	74.31–75.68
	3	96	75.85 ± 0.21	2.05	68.69–81.34	75.43–76.26
	4	35	75.27 ± 0.36	2.11	67.12–78.19	74.54–76.00
	5	48	78.02 ± 0.33	2.30	71.55–80.94	77.35–78.68

THI = Temperature–humidity index, CI = Confidence interval, n = Number of flocks, SD = Standard deviation, SEM = Standard error of the mean.

Across farms, mean maximum temperatures ranged from 33.52–35.47°C on day 1 and 27.93–29.16°C at slaughter (Table 4), with Farm 2 recording the highest values at both time points (35.47°C and 29.16°C). Mean minimum temperatures ranged from 31.67–33.63°C on day 1 and 24.99–27.10°C at slaughter. Mean ΔT ranged from 0.51 to 4.99°C, indicating substantial differences in thermal stability among farms. Farms 2 and 4 consistently showed higher temperatures, particularly during the early rearing period (days 1–21), with Farm 2 recording the highest day-1 temperature (35.47°C), followed by Farm 4 (34.83°C). The Cobb 500 Broiler Management Guide recommends air temperatures of 32–33°C at placement, decreasing to 27–29°C by days 8–14 and 25–27°C by days 15–21 [3]. In this study, all farms recorded temperatures 3–5°C above these targets, especially in Farms 2 and 4. High temperatures persisted beyond placement throughout brooding and early starter phases, resulting in prolonged exposure during critical skeletal development stages.

**Figure 2:**
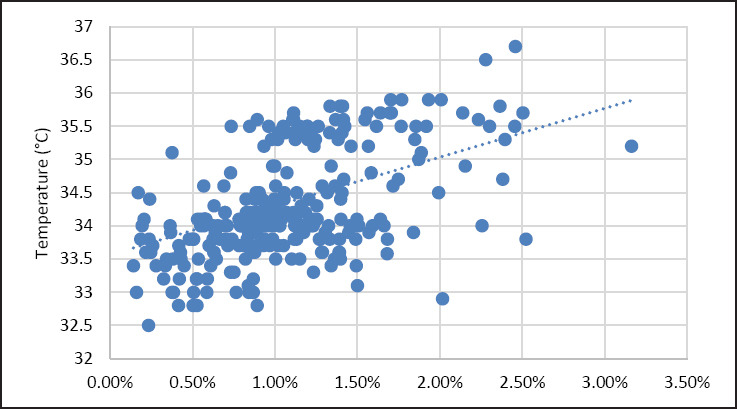
Relationship between the highest temperature on day 1 and lameness incidence. Figure 2 illustrates the association between the highest ambient temperature on day 1 and flock-level lameness incidence. Each point represents one flock, and the dotted line indicates the fitted linear regression. A clear positive trend is observed, indicating that higher brooding temperatures were associated with increased lameness prevalence. Most observations clustered between 33.0°C and 35.5°C, while flocks exposed to temperatures above 35.5°C tended to exhibit higher lameness levels. This pattern supports the significant positive correlation observed in the statistical analysis.

Correlation analysis revealed significant age-dependent links between temperature and lameness (Table 5). As shown in Figure 2, the highest daily temperature was strongly correlated with lameness on day 1 (r = 0.54, p < 0.0001), with weaker but still significant associations on days 7, 14, and 21, which became non-significant later. These results suggest that higher temperatures during brooding and early starter phases increase risk for locomotor disorders in tropical commercial settings.

Lowest temperature showed a dynamic pattern. Positive correlations with lameness were observed on days 1 and 7 (p < 0.001) and remained significant after FDR adjustment (Supplementary Table 4). After FDR correction, the day 21 association was not significant, whereas the negative correlation at day 28 remained significant (q = 0.03040), indicating a stronger late-stage effect. ΔT showed stage-specific patterns: a negative correlation on day 7 (r = −0.36; q = 0.00025) shifted to positive from day 14 onward, remaining significant at day 28 (q = 0.00871), suggesting that large day–night temperature swings may increase lameness risk.

THI was calculated to reflect the combined thermal load. THI remained relatively high throughout the production cycle, particularly during early and mid-growth (Table 6). Based on Cobb-Vantress guidelines, reference THI values are approximately 74–81 during brooding and 67–72 during finishing. Observed THI values were generally higher than these references, indicating sustained exposure to elevated heat–humidity load that may contribute to heat stress and impaired musculoskeletal development. Similar associations between high THI, reduced performance, and increased leg disorders have been reported in tropical production systems, supporting the broader relevance of these findings.

Table 7 shows no significant association between high-temperature THI and lameness (p > 0.05), whereas low-temperature THI showed a notable negative correlation during days 14–28 (r = −0.22 to −0.26, p < 0.01), emphasizing the importance of humidity and temperature effects at night. Early skeletal and thermoregulatory immaturity, combined with rapid muscle growth after day 14, increases broilers’ sensitivity to environmental conditions. Temperatures often exceeded Cobb-Vantress recommendations, particularly on Farms 2 and 4 during days 8–14, potentially impairing bone development and increasing the risk of leg disorders. Early-life heat exposure, unstable diurnal temperatures, and unfavorable nighttime THI were linked to lameness, with day 1 temperature and day 14 light intensity serving as practical early-warning indicators.

**Table 7:** Pearson’s correlation coefficients between THI at the highest and lowest temperatures and total lameness incidence across all farms and rearing days.

Variable	Day	n	r	95% CI	p-value
THI at highest temperature	1	283	−0.00	−0.12 to 0.11	0.98
	7	293	−0.11	−0.23 to 0.01	0.05
	14	295	−0.01	−0.21 to 0.11	0.91
	21	296	0.03	−0.08 to 0.15	0.57
	28	296	−0.04	−0.15 to 0.07	0.50
	35	296	0.00	−0.11 to 0.12	0.94
	Slaughter	296	0.07	−0.05 to 0.18	0.26
THI at lowest temperature	1	275	−0.04	−0.15 to 0.08	0.55
	7	294	0.04	−0.08 to 0.15	0.52
	14	292	−0.26	−0.38 to −0.15	< 0.0001
	21	294	−0.23	−0.34 to −0.12	< 0.0001
	28	292	−0.22	−0.32 to −0.10	0.0002
	35	294	−0.05	−0.16 to 0.07	0.41
	Slaughter	293	−0.18	−0.29 to −0.07	0.0019

THI = Temperature–humidity index, CI = Confidence interval, n = Number of flocks, r = Pearson’s correlation coefficient.

### Light intensity and locomotor health

Light intensity varied substantially across the five farms and frequently exceeded the recommended management levels for broilers (Table 8). During the brooding period (days 1–7), Farms 2 and 4 recorded the highest mean light intensities (43.69 and 48.96 lux, respectively), whereas Farms 1, 3, and 5 maintained lower levels between 22.72 and 34.17 lux. From day 14 onwards, Farms 2 and 4 consistently sustained high light levels (>50 lux). In contrast, Farm 3 maintained the lowest levels throughout the production cycle, averaging 31–32 lux. By slaughter day, Farms 1, 2, and 4 still had light intensities exceeding 59 lux, while Farm 3 and Farm 5 remained around 32–33 lux. Notably, Farms 1, 3, and 5, which maintained comparatively lower light intensities, also exhibited lower lameness incidence, further supporting the link between excessive light exposure and leg health problems.

**Table 8:** Descriptive statistics of light intensity in broiler houses across farms.

Day	Farm	n	Mean ± SEM (lux)	SD	Min–max (lux)	95% CI (lux)
1	1	60	22.72 ± 0.14	1.09	20–26	22.43–23.00
	2	60	43.69 ± 0.73	5.66	34.3–53.4	42.23–45.15
	3	76	31.24 ± 0.38	3.32	25–36	30.48–32.00
	4	36	48.96 ± 1.74	10.45	30.2–64.6	45.43–52.50
	5	48	34.17 ± 0.57	3.98	20–38	33.01–35.32
7	1	60	25.45 ± 0.54	4.22	21–45	24.36–26.54
	2	60	58.32 ± 0.85	6.61	50–78.2	56.61–60.03
	3	76	31.72 ± 0.37	3.21	25–38	31.00–32.46
	4	36	52.86 ± 1.04	6.25	42.3–62.8	50.75–54.97
	5	48	34.08 ± 0.51	3.54	20–38	33.06–35.11
14	1	60	28.17 ± 0.86	6.70	22–50	26.44–29.90
	2	60	58.45 ± 0.71	5.50	50–71	57.03–59.87
	3	76	31.72 ± 0.36	3.12	25–36	31.01–32.44
	4	36	53.56 ± 1.01	6.08	41.3–68	51.50–55.61
	5	48	34.58 ± 0.58	4.03	20–39	33.41–35.75
21	1	60	34.58 ± 1.17	9.03	22–52	32.25–36.91
	2	60	59.43 ± 0.81	6.31	51.5–78	57.80–61.05
	3	72	31.61 ± 0.37	3.14	26–38	30.87–32.35
	4	36	55.99 ± 1.01	6.05	44.2–66.4	53.94–58.04
	5	48	34.44 ± 0.61	4.20	20–40	33.22–35.66
28	1	60	43.27 ± 1.60	12.40	25–65	40.06–46.47
	2	60	61.14 ± 0.58	4.52	52.1–65	59.97–62.31
	3	60	32.25 ± 0.37	2.84	25–38	31.52–32.98
	4	36	58.52 ± 0.91	5.45	44.7–65.5	56.67–60.36
	5	48	34.02 ± 0.54	3.71	21–37	32.94–35.10
35	1	60	53.98 ± 1.58	12.26	25–75	50.82–57.15
	2	60	62.00 ± 0.66	5.10	50.3–67.2	60.69–63.31
	3	56	32.27 ± 0.42	3.14	25–38	31.43–33.11
	4	36	57.09 ± 0.84	5.06	48.4–65	55.38–58.80
	5	48	35.31 ± 0.57	3.97	22–38	34.16–36.46
Slaughter	1	60	66.82 ± 1.72	13.29	25–95	63.38–70.25
	2	60	63.23 ± 0.67	5.19	51.1–66.9	61.89–64.57
	3	56	32.68 ± 0.36	2.73	25–38	31.95–33.41
	4	36	59.81 ± 0.71	4.29	49.5–67.6	58.36–61.26
	5	48	33.27 ± 0.51	3.51	21–39	32.25–34.29

CI = Confidence interval, n = Number of flocks, SD = Standard deviation, SEM = Standard error of the mean.

**Figure 3:**
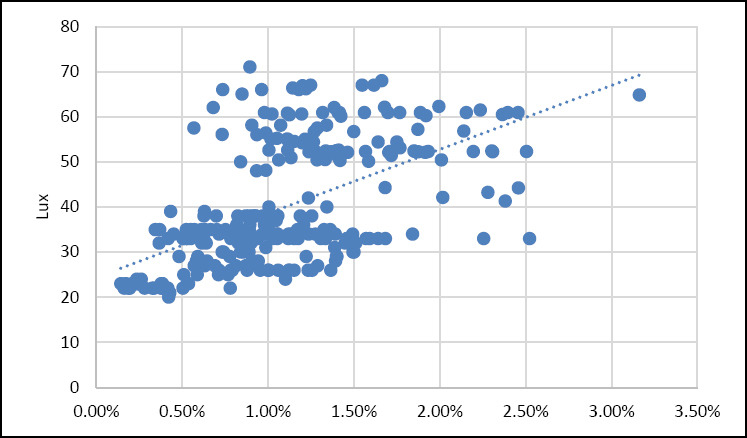
Relationship between light intensity on day 14 and lameness incidence. Figure 3 shows the relationship between light intensity on day 14 and lameness incidence. The scatterplot demonstrates a strong positive association, with increasing light intensity corresponding to higher lameness prevalence. Two main clusters are evident, representing moderate lighting regimes of approximately 25–35 lux and high lighting regimes of >50 lux. Flocks exposed to higher light intensities consistently exhibited elevated lameness levels. The regression line confirms this trend, consistent with the strong correlation and multivariable regression results.

Correlation analysis confirmed that light intensity was strongly associated with lameness incidence across all rearing stages (Table 9). This relationship is illustrated in Figure 3, showing that higher light intensity on day 14 was associated with increased lameness incidence. The strongest correlations were observed at day 14 (r = 0.56, p < 0.0001) and day 21 (r = 0.52, p < 0.0001), coinciding with the period of rapid skeletal mineralization and subsequent accelerated muscle growth. Even at day 1, light intensity was positively correlated with lameness (r = 0.41, p < 0.0001), suggesting that inappropriate lighting conditions from the earliest stages can predispose broilers to locomotor disorders. This period corresponds to the transition phase in which skeletal mineralization may lag behind rapid muscle accretion, making birds particularly vulnerable to excessive mechanical loading induced by high activity levels under intense illumination.

**Table 9:** Pearson’s correlation coefficients between light intensity and total lameness incidence across all farms and rearing days.

Variable	Day	n	r	95% CI	p-value
Light intensity	1	270	0.41	0.31–0.50	< 0.0001
	7	274	0.52	0.43–0.60	< 0.0001
	14	270	0.56	0.47–0.64	< 0.0001
	21	272	0.52	0.43–0.60	< 0.0001
	28	264	0.52	0.42–0.61	< 0.0001
	35	272	0.33	0.22–0.43	< 0.0001
	Slaughter	270	0.15	0.03–0.27	0.012

CI = Confidence interval, n = Number of flocks, r = Pearson’s correlation coefficient.

These findings deviate markedly from the Cobb Genetics guidelines [19], which recommend a minimum of 25 lux during the first 0–7 days to stimulate early feed intake, followed by a gradual reduction to 5–10 lux from day 7 onwards to balance welfare and performance. In addition, the variation in light intensity across the floor should not exceed 20%. The persistently high light levels in Farms 2 and 4, particularly beyond day 14, exceeded these recommendations and likely contributed to higher lameness incidence. During days 8–21, measured light intensities in Farms 2 and 4 frequently reached 50–59 lux, exceeding recommended levels by approximately two- to four-fold [3]. Such prolonged exposure to excessive illumination during periods of rapid skeletal growth may have amplified mechanical loading on immature leg structures.

Recent research provides biological support for the observed association between light environment and leg health in broilers. Higher light intensities (>30–50 lux) have been shown to increase bird activity, such as walking, foraging, and competitive interactions, thereby elevating mechanical loading on immature skeletal structures and increasing the likelihood of locomotor problems, particularly in fast-growing strains [20, 21]. Prolonged or continuous light exposure further reduces opportunities for rest and compromises cartilage turnover and bone remodeling, especially during periods of rapid weight gain [5, 20]. Conversely, suboptimal light levels during brooding (<20 lux) may limit feed- and water-locating behavior, delay early skeletal development, and increase long-term susceptibility to lameness [5, 21]. In this study, Farms 2 and 4 exhibited characteristics consistent with excessive light exposure, while Farm 1 occasionally approached suboptimal levels during brooding, aligning with these documented risks.

Overall, the results demonstrate that inappropriate light-intensity management, particularly excessive illumination, during both the early brooding period (days 0–7) and the critical skeletal development phase (days 14–21) was strongly associated with increased lameness incidence. Recent studies show that high light intensity elevates activity levels and mechanical loading on immature leg structures, while insufficient dark periods reduce resting opportunities and impair skeletal recovery [20, 21]. These findings emphasize the importance of adopting appropriate lighting programs, including reducing light intensity after the first week and ensuring structured dark periods to support rest, bone remodeling, and overall locomotor health. Aligning farm practices with breeder recommendations is therefore essential to minimizing leg strain and improving broiler welfare.

### Ammonia concentration and late-stage risk

Ammonia concentrations gradually increased with bird age across all farms (Table 10). According to EFSA and international welfare guidelines, ammonia concentrations above 15.00 ppm are associated with respiratory irritation, reduced performance, and compromised welfare. During the first two weeks, mean ammonia levels remained low. At day 7, concentrations ranged between 3.15 and 4.35 ppm, and by day 14 between 4.57 and 6.00 ppm, all well below the recommended threshold of 10.00–15.00 ppm [3].

**Table 10:** Descriptive statistics of ammonia concentration in broiler houses across farms.

Day	Farm	n	Mean ± SEM (ppm)	SD	Min–max (ppm)	95% CI (ppm)
7	1	60	3.15 ± 0.11	0.84	2–5	2.93–3.37
	2	60	3.55 ± 0.07	0.53	3–5	3.41–3.69
	3	82	3.63 ± 0.14	1.24	1–6	3.36–3.91
	4	34	4.35 ± 0.16	0.92	2–7	4.03–4.67
	5	48	3.31 ± 0.10	0.72	2–4	3.10–3.52
14	1	60	4.57 ± 0.15	1.18	2–8	4.26–4.87
	2	60	5.60 ± 0.08	0.62	3–6	5.44–5.76
	3	82	5.70 ± 0.19	1.70	4–9	5.32–6.07
	4	34	6.00 ± 0.13	0.79	4–8	5.73–6.27
	5	48	4.69 ± 0.09	0.66	3–5	4.50–4.88
21	1	60	6.30 ± 0.16	1.27	3–9	5.97–6.63
	2	60	7.12 ± 0.08	0.58	6–8	6.97–7.27
	3	82	7.15 ± 0.25	2.22	5–11	6.66–7.63
	4	34	7.06 ± 0.15	0.89	5–9	6.75–7.37
	5	48	6.21 ± 0.12	0.82	4–7	5.97–6.45
28	1	60	7.90 ± 0.16	1.26	4–10	7.57–8.23
	2	60	11.95 ± 0.13	1.02	11–16	11.69–12.21
	3	62	9.52 ± 0.28	2.18	6–13	8.96–10.07
	4	34	11.09 ± 0.12	0.71	10–12	10.84–11.34
	5	48	10.67 ± 0.28	1.97	5–12	10.09–11.24
35	1	60	9.73 ± 0.19	1.49	6–12	9.35–10.12
	2	60	13.95 ± 0.16	1.27	12–16	13.62–14.28
	3	50	11.96 ± 0.17	1.18	10–14	11.63–12.29
	4	34	12.35 ± 0.27	1.55	9–15	11.81–12.90
	5	48	11.79 ± 0.25	1.76	7–13	11.28–12.30
Slaughter	1	60	12.03 ± 0.15	1.13	8–15	11.74–12.33
	2	60	16.15 ± 0.08	0.63	15–17	15.99–16.31
	3	50	15.34 ± 0.12	0.87	13–17	15.09–15.59
	4	34	14.97 ± 0.16	0.90	12–16	14.66–15.29
	5	48	15.67 ± 0.17	1.19	10–16	15.32–16.01

NH₃ = Ammonia, CI = Confidence interval, n = Number of flocks, SD = Standard deviation, SEM = Standard error of the mean.

From day 21 onwards, ammonia concentrations progressively increased, reaching approximately 6.21–7.15 ppm. By day 28, mean values ranged from 7.90 to 11.95 ppm, with several farms exceeding 10.00 ppm. At day 35, concentrations further increased to 9.73–13.95 ppm, approaching the upper welfare threshold. By slaughter day, mean ammonia levels reached 16.15 ppm in Farm 2 and 15.34 ppm in Farm 3, surpassing the 15.00 ppm welfare threshold, while Farm 4 approached this limit (14.97 ppm). These findings indicate that although ammonia remained within acceptable limits during early rearing, late-stage accumulation exposed birds to suboptimal air quality conditions.

**Figure 4:**
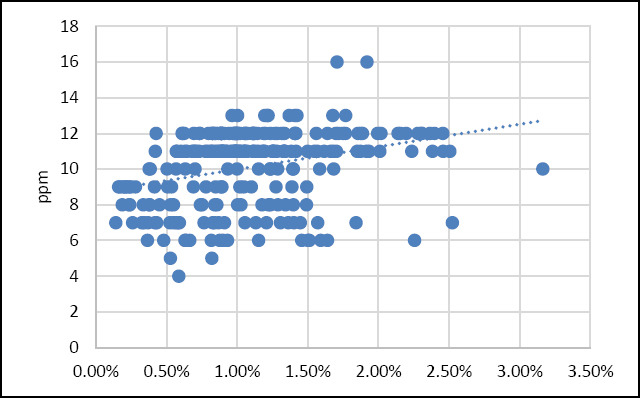
Relationship between ammonia concentration on day 28 and lameness incidence. Figure 4 presents the association between ammonia concentration on day 28 and lameness incidence. Although a weak positive trend is visible, the data show substantial variability, particularly at moderate ammonia levels of 8–12 ppm. Several flocks with similar ammonia concentrations exhibited markedly different lameness outcomes. The regression line indicates only a modest association, which is consistent with the non-significant effect of ammonia in the final multivariable model.

The correlation analysis (Table 11) further supported these observations. Ammonia concentrations showed no significant relationship with lameness incidence during the early stages (day 7–21), with correlation coefficients close to zero (r = 0.06 to 0.03, p > 0.3). From day 28 onwards, however, the relationship became evident, as illustrated in Figure 4. Positive and significant correlations were observed (day 28: r = 0.31, p < 0.0001; day 35: r = 0.41, p < 0.0001; slaughter day: r = 0.39, p < 0.0001). This suggests that ammonia accumulation beyond 10 ppm during the late growing period was associated with increased lameness incidence.

**Table 11:** Pearson’s correlation coefficient between ammonia concentration and total lameness incidence across all farms and rearing days.

Variable	Day	n	r	95% CI	p-value
Ammonia concentration	7	284	0.06	−0.06 to 0.17	0.3555
	14	284	−0.01	−0.13 to 0.10	0.8163
	21	284	0.03	−0.08 to 0.15	0.5674
	28	264	0.31	0.20–0.42	< 0.0001
	35	252	0.41	0.31–0.51	< 0.0001
	Slaughter	252	0.39	0.28–0.49	< 0.0001

CI = Confidence interval, n = Number of flocks, r = Pearson’s correlation coefficient.

The biological mechanisms underlying this relationship are well-documented. High ammonia exposure can cause respiratory irritation and lung damage, leading to hypoxia, which indirectly impairs skeletal development [3, 22]. Elevated ammonia levels also compromise litter quality, predisposing birds to footpad dermatitis and increased joint pressure, which subsequently contribute to locomotor impairment [3, 23–25]. Additionally, poor air quality due to high ammonia levels can increase susceptibility to bacterial infections, such as bacterial chondronecrosis with osteomyelitis (BCO), thereby exacerbating leg health issues [22]. However, detailed litter quality parameters were not systematically evaluated. Although litter quantity per bird was similar across farms, variation in litter condition may have affected ammonia accumulation and leg health, potentially confounding the interpretation of the results. Taken together, these results indicate that ammonia did not pose a major risk for lameness during the brooding and early rearing stages, as concentrations remained below the critical threshold of 10 ppm. However, progressive ammonia accumulation in the later stages (>28 days) significantly increased the risk of lameness, particularly in Farms 2, 4, and 5, where ammonia levels exceeded or approached 15 ppm. These findings underscore the importance of maintaining effective ventilation and litter management throughout the production cycle to prevent late-stage ammonia buildup and its detrimental effects on leg health.

Prolonged exposure to suboptimal temperatures, excessive light intensity, and elevated ammonia levels may increase economic losses through reduced growth, increased culling, and greater carcass rejection due to locomotor disorders. After Benjamini–Hochberg FDR adjustment, key associations (day 1 Tmax, day 7 ΔT, day 14 light intensity, and late-stage ammonia) remained significant (Supplementary Table 4), supporting the robustness of these relationships.

### Multivariable regression model

The stepwise selection procedure (Supplementary Table 3) identified early thermal conditions and mid-cycle light intensity as candidate predictors of lameness. However, to maintain biological interpretability and avoid multicollinearity among correlated environmental variables, a final multivariable model was constructed a priori using five predictors: highest temperature on day 1, diurnal temperature difference on day 7, light intensity on day 14, and ammonia concentrations on days 28 and 35 (Table 12). The estimated regression equation was:

Lameness (%) = −0.137 + 0.0122 T_maxD1 − 0.0829 ΔT_D7 + 0.0197 L_D14 + 0.0381 NH_3__D28 − 0.0234 NH_3__D35

where T_maxD1 is the highest temperature on day 1 (°C), ΔT_D7 is the diurnal temperature on day 7 (°C), L_D14 is the light intensity on day 14 (lux), and NH_3__D28 and NH_3__D35 are ammonia concentrations on days 28 and 35 (ppm).

**Table 12:** Multiple regression model evaluating environmental predictors of lameness (%) in commercial broilers.

Variable	β (Estimate)	SE	t	p-value	VIF
Intercept	−0.137	0.679	−0.20	0.840	–
Highest temperature (Day 1)	0.0122	0.0204	0.60	0.551	1.24
Diurnal temperature (Day 7)	−0.0829	0.0264	−3.14	0.0019	1.28
Light intensity (Day 14)	0.0197	0.00244	8.07	< 0.0001	1.58
Ammonia concentration (Day 28)	0.0381	0.0266	1.43	0.153	4.36
Ammonia concentration (Day 35)	−0.0234	0.0276	−0.85	0.399	4.62

The final regression model incorporated five environmental predictors selected a priori based on biological relevance and previous correlation analysis. Light intensity on day 14 and diurnal temperature on day 7 were statistically significant predictors of lameness, whereas highest temperature on day 1 and ammonia concentration on days 28 and 35 were not statistically significant in the multivariable model.

R² = 0.429, adjusted R² = 0.416, F(5, 222) = 33.38, p < 0.0001, root MSE = 0.400, Durbin–Watson = 0.98, n = 228.

SE = Standard error, VIF = Variance inflation factor.

**Figure 5:**
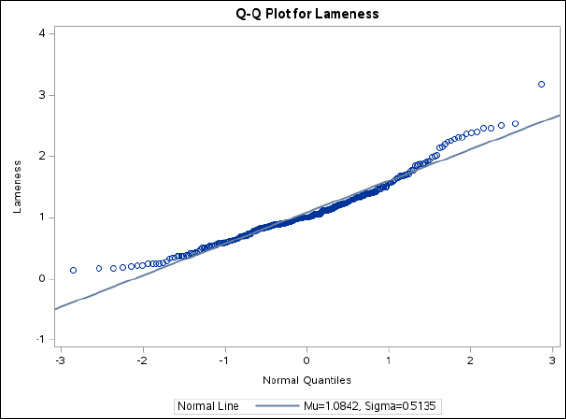
Quantile–quantile plot of flock-level lameness (%) showing approximate normal distribution of residuals. Figure 5 presents the normal quantile–quantile plot for flock-level lameness percentage. Each point represents one flock plotted against the theoretical normal distribution quantiles. Most observations closely follow the reference line, indicating that the lameness data approximate a normal distribution. Minor deviations are observed at the upper tail, where a few flocks exhibited relatively higher lameness incidence. However, these deviations were limited and did not substantially violate normality assumptions. This pattern supports the suitability of applying the General Linear Model and linear regression analyses to the dataset.

The final multivariable regression model (Table 12) explained 42.9% of the variation in flock-level lameness percentage (R² = 0.429; adjusted R² = 0.416; F(5,222) = 33.38, p < 0.0001; Root MSE = 0.400 % units). A greater diurnal temperature difference on day 7 (β = −0.0829, p = 0.0019) was linked to less lameness, while higher light intensity on day 14 (β = 0.0197, p < 0.0001) predicted more lameness. Maximum temperature on day 1 and ammonia levels on days 28 and 35 were not significant predictors. Variance inflation factors (VIFs) ranged from 1.24 to 4.62, indicating no significant multicollinearity. The Durbin–Watson statistic of 0.98 suggested mild positive autocorrelation. Q–Q plots in Figure 5 show near-normal residuals, with minor upper-tail deviations unlikely to impact model validity.

Although the final models explained a substantial proportion of the variation in lameness, a considerable amount of unexplained variability remained. Potential interaction effects between early thermal stress, light intensity, and late-stage air quality were not formally tested in the present model and warrant further investigation in future studies. Other unmeasured factors such as within-strain genetic differences, flock-level variation in stocking density, diet formulation (e.g., calcium, phosphorus, and vitamin D levels), litter management routines, and infectious causes of leg disorders may also have contributed to lameness but were not available in the present dataset. Therefore, the associations reported here should be interpreted as the environmental component of a broader multifactorial process rather than as definitive causal effects.

These results suggest that a practical farm-level risk framework using early-life maximum temperature (day 1), diurnal temperature (day 7), mid-cycle light intensity (day 14), and late-stage ammonia may support proactive management in tropical closed-house systems. However, the framework should be validated in additional flocks and tested for interaction effects before use as a predictive tool.

## CONCLUSION

This study demonstrated that lameness in commercial Cobb 500 broilers raised under tropical closed-house conditions was associated with age-specific environmental stressors. The median lameness incidence was 1.00%, with most flocks remaining below 1.5%; however, significant farm-level variation was observed. Early-life thermal stress, particularly higher day 1 temperature, was positively associated with lameness, while day 7 diurnal temperature variation and day 14 light intensity emerged as key predictors in the final multivariable model. Late-stage NH_3_ accumulation after day 28 was also positively correlated with lameness in univariable analysis, indicating that air quality becomes increasingly important as birds approach market age.

The findings have direct practical implications for tropical broiler production. Maintaining brooding temperatures near breeder recommendations, avoiding excessive light intensity during the rapid skeletal development phase, and improving ventilation and litter management during late growth may reduce the risk of lameness. In particular, monitoring day 1 temperature, day 7 diurnal temperature variation, day 14 light intensity, and late-stage NH_3_ concentration may provide a practical early-warning framework for farm managers and veterinarians.

The major strength of this study is its large-scale commercial dataset, including 296 flocks and more than 11.45 million broilers across five closed-house farms managed under a standardized integrator system. This design reduced variation related to feeding, genetics, health management, and biosecurity, allowing clearer evaluation of environmental risk factors under real-world tropical production conditions.

However, the study also had limitations. The lameness diagnosis was based on clinical culling records without postmortem confirmation, and interobserver reliability was not formally assessed. Environmental measurements were mainly collected at central house locations, and within-house variation, litter condition, detailed infectious causes, and economic outcomes were not fully evaluated. The regression model explained approximately 43% of the variation in lameness, indicating that additional biological, management, and health-related factors likely contributed to the observed outcomes.

Future studies should validate these findings in independent commercial flocks and across different seasons, stocking densities, ventilation systems, and genetic lines. Further research should also include multi-point environmental sensing, litter quality assessment, postmortem confirmation of leg lesions, economic impact analysis, and mixed-effects or longitudinal modeling to test interactions among early thermal stress, lighting, and late-stage air quality.

In conclusion, lameness in tropical closed-house broiler systems is a multifactorial condition strongly influenced by time-specific environmental exposure. Optimizing early thermal management, controlling mid-growth light intensity, and preventing late-stage NH_3_ buildup may improve broiler welfare, reduce culling losses, and support more sustainable commercial poultry production.

## DATA AVAILABILITY

The supplementary data can be made available from the corresponding author upon request.

## AUTHORS’ CONTRIBUTIONS

PN and WL: Conceived and designed the study. PN: Data collection, preliminary data organization, and drafting of the original manuscript. PN and CR: Data analysis and statistical interpretation. CB: Supervision of the research, scientific guidance, and critical revision of the manuscript. PN, WL, CR, and CB: Review and editing of the manuscript. All authors have read and approved the final version of the manuscript.
